# Host phylogeny and environment shape the diversity of salamander skin bacterial communities

**DOI:** 10.1186/s42523-023-00271-7

**Published:** 2023-10-13

**Authors:** S. Ramírez-Barahona, F. M. González-Serrano, E. Martínez-Ugalde, A. Soto-Pozos, G. Parra-Olea, E. A. Rebollar

**Affiliations:** 1 Departamento de Botánica, Instituto de Biología, Universidad Nacional Autónoma de México, Ciudad de México, Mexico; 2 Centro de Ciencias Genómicas, Universidad Nacional Autónoma de México, Cuernavaca, Morelos, Mexico; 3 Departamento de Zoología, Instituto de Biología, Universidad Nacional Autónoma de México, Ciudad de México, Mexico

## Abstract

The composition and diversity of animal-associated microbial communities are shaped by multiple ecological and evolutionary processes acting at different spatial and temporal scales. Skin microbiomes are thought to be strongly influenced by the environment due to the direct interaction of the host’s skin with the external media. As expected, the diversity of amphibian skin microbiomes is shaped by climate and host sampling habitats, whereas phylogenetic effects appear to be weak. However, the relative strength of phylogenetic and environmental effects on salamander skin microbiomes remains poorly understood. Here, we analysed sequence data from 1164 adult salamanders of 44 species to characterise and compare the diversity and composition of skin bacteria. We assessed the relative contribution of climate, host sampling habitat, and host phylogeny to the observed patterns of bacterial diversity. We found that bacterial alpha diversity was mainly associated with host sampling habitat and climate, but that bacterial beta diversity was more strongly associated with host taxonomy and phylogeny. This phylogenetic effect predominantly occurred at intermediate levels of host divergence (0–50 Mya). Our results support the importance of environmental factors shaping the diversity of salamander skin microbiota, but also support host phylogenetic history as a major factor shaping these bacterial communities.

**Supplementary Information:**

The online version contains supplementary material available at 10.1186/s42523-023-00271-7.

## Introduction

Throughout evolutionary history microbial communities have established multiple symbiotic interactions with animals [1–3]. Different animal organs (e.g., gut, skin) represent distinct and unique microhabitats that enable colonisation of different microbial taxa, some of which establish mutualistic relations with their host [4–6]. The composition and diversity of these animal-associated microbial communities (microbiomes) are shaped by multiple ecological and evolutionary processes acting at different spatial and temporal scales [7–10]. In many animal groups, closely related host species harbour microbiotas with similar composition [1, 4, 6], which can be attributed to host phylogenetic effects. However, microbiome similarities are also shaped by host-associated factors, such as immunity and diet [11, 12], and environmental factors such as microhabitats and climate [4, 5].

The relative influence of distinct factors depends on the nature of the animal-microbial interaction (e.g., organ system) and the physiological and immunological characteristics of the host [3, 6, 11, 12]. Unlike gut microbiomes, skin microbiomes are thought to be more strongly influenced by environmental factors due to the direct interaction of the host’s skin with the external media. For instance, the skin microbiota of amphibians is largely influenced by large-scale climatic factors (e.g., precipitation, temperature) and microhabitats (e.g., arboreal, terrestrial, or aquatic lifestyles) [9, 13, 14]. Also, host developmental transitions [15] linked to immunological changes [16], innate immunity, and host genetic diversity (e.g., at the major histocompatibility complex [17]) play a major role in shaping these microbial communities [11, 18]. Skin microbiomes in amphibians function as an extension of the host immune system [19] and could partially explain the variability in susceptibility of amphibian species to emerging pathogens [14].

The role of host phylogeny in shaping the skin microbiota of amphibians remains unclear, but evidence suggests host-phylogenetic history has a significant, albeit weak, effect in shaping skin microbiomes [9, 12]. However, several climatic factors likely carry a phylogenetic signal due to niche conservatism across the amphibian tree of life, which could be masking underlying host-phylogenetic effects. Furthermore, animal-microbiome interactions vary through evolutionary time [4, 6] and the magnitude of phylogenetic effects on skin microbiome assemblages depend on the evolutionary scale being analysed for both hosts (e.g., species within genera) and microbes (e.g., bacterial orders) [4, 6]. Indeed, several studies have found substantial differences in skin microbial diversity among distinct amphibian families, genera, species, and sub-species [9, 20–23] and have suggested a more prominent effect of host phylogeny on skin microbiomes.

The contribution of different environmental and host-associated factors in shaping the skin microbiomes across the amphibian tree of life is not yet fully explored. Most studies addressing these questions have focused on frogs and toads (Anura) [9, 12, 14, 18, 24] and less attention has been paid to salamanders (Caudata) [15, 22, 23, 25–27]. A global analysis of amphibian microbiomes included over 200 anuran species but less than 30 salamander species [9]. Salamanders have two key biological features that allow for a comprehensive assessment of the relative contribution of different factors shaping skin microbiomes: (i) adult salamanders can be either fully terrestrial (e.g., many plethodontids), fully aquatic (e.g., axolotls, hellbenders) or a combination of both where adults move into aquatic habitats for reproduction but juveniles are terrestrial (e.g., newts), whereas only a few frog species are fully aquatic as adults [28, 29]; and (ii) salamanders are geographically and climatically more restricted than frogs and toads, mostly inhabiting more temperate climates in the Northern hemisphere [28, 29].

To address this knowledge gap, here we assess the relative contribution of host sampling habitat, climate, elevation, and host phylogenetic relationships to the diversity and structure of skin bacterial communities in salamanders. We compiled available 16S rRNA sequence data of salamander skin bacterial communities, including newly generated data by our working group, and used sampling habitat and climatic data to test for host-associated and environmental correlations with bacterial diversity. Finally, we constructed a dated phylogeny for extant salamanders to test for evidence of a phylogenetic signal in the composition of skin bacteria.

## Results

### Host habitat and taxonomy influence skin bacterial diversity

**Fig. 1:**
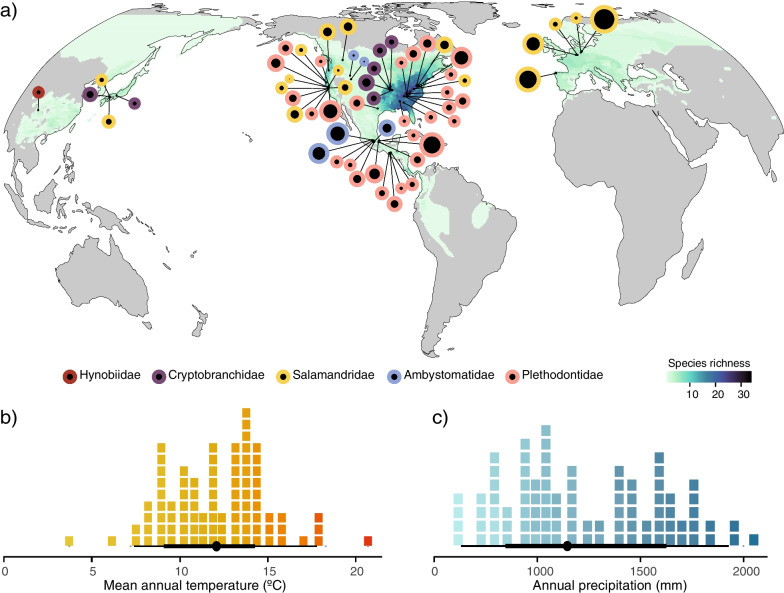
Geographic and climatic distribution of localities sampled for salamander skin bacterial communities. **a** Geographic distribution of sampling localities coloured by salamander family. The size of circles is proportional to the number of samples per geographic location. The colour scale on the map depicts salamander species diversity at a 10 × 10 km resolution obtained from https://biodiversitymapping.org. The location of samples from captive salamanders (representing two species) is not shown. **b** Annual temperature and **c** annual precipitation data distribution of sampling localities

**Fig. 2:**
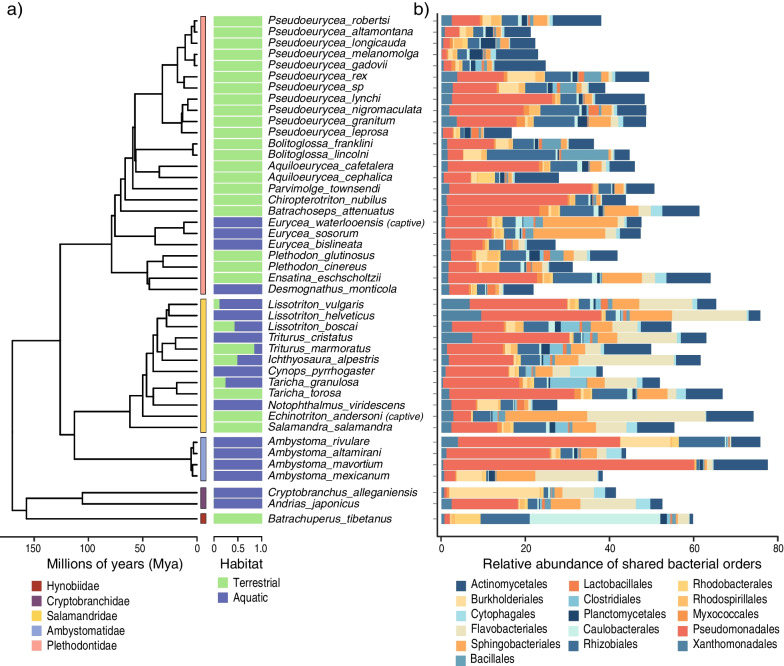
Salamander phylogeny, host sampling habitat, and relative abundance of the shared skin bacterial orders. **a** Species-level dated phylogeny for salamanders showing phylogenetic relationships and divergence times, and the proportion of samples taken from different habitats (aquatic or terrestrial). The tree represents a ‘pruned’ version of the complete species-level phylogeny that includes species with skin microbiome data (see Additional file 1: Fig. S1). **b** Relative abundances of the 16 shared bacterial orders for each salamander host species. Orders are arranged from left to right in stacked graphs and from upper-left to bottom-right in the legend

We used 16S rRNA amplicon sequence data from 21 datasets to compile 1,164 adult salamander samples, across 87 localities, and characterise the diversity and composition of skin bacterial communities in 44 host species; two host species are represented only by captive individuals (*Echinotriton andersonii* and *Eurycea waterlooensis*). Most of the sampled localities lay within centres of salamander species richness, especially in North America (Fig. 1). The 44 host species represent five out of the ten currently recognized salamander families (Fig. 2; Additional file 1: Fig. S1), but the majority of sampled species belong to the two largest families Plethodontidae and Salamandridae. Most species are represented by individuals sampled from either terrestrial or aquatic habitats, with the exception of five species of Salamandridae in which individuals were sampled from both terrestrial and aquatic habitats (Fig. 2a).

After bioinformatic processing of the data we obtained Amplicon Sequence Variants (ASVs) that were taxonomically assigned and focused our analyses on bacterial order and family levels (see Methods). We found 223 bacterial orders and 453 bacterial families across all salamander samples. We identified 25 bacterial orders and 23 families shared among all salamander species, irrespective of host sampling habitat or family (Additional files 9 and 10); sixteen shared orders were successfully assigned to recognized bacterial taxa and were used for subsequent analyses. These shared orders comprised 16.6–77.4% of the relative abundances of ASVs across all host species (Fig. 2b). Thirteen bacterial orders had a median prevalence > 80% across host species and five of these orders (Rhizobiales, Sphingobacteriales, Pseudomonadales, Xanthomonadales, and Actinomycetales) kept high levels of prevalence (> 80%) in three quarters or more of the sampled salamander species (Additional file 1: Fig. S2). However, other shared bacterial orders had a more varying prevalence among host species (e.g., Myxococcales).

By implementing Linear Discriminant Analysis Effect Size (LefSe) we identified bacterial orders whose relative abundances explain differences among samples from distinct sampling habitats or salamander families. We found 91 bacterial orders with statistically significant differences between the terrestrial and aquatic sampling habitats, but only 69 could be taxonomically assigned to named orders: 44 with higher abundances in terrestrial habitats and 25 with higher abundances in aquatic habitats (Additional file 11). In turn, we found 18 bacterial orders with differences among host families, out of which 13 could be taxonomically assigned to named orders. All but one of the bacterial orders with differences among families were also identified as differentially abundant among host sampling habitats (Additional file 12). Overall, we found evidence of different abundance profiles solely by sampling habitat in 74 bacterial orders (out of 91), whereas only one order (out of 18) showed higher abundances by host family, specifically in the Cryptobranchidae family.

**Fig. 3:**
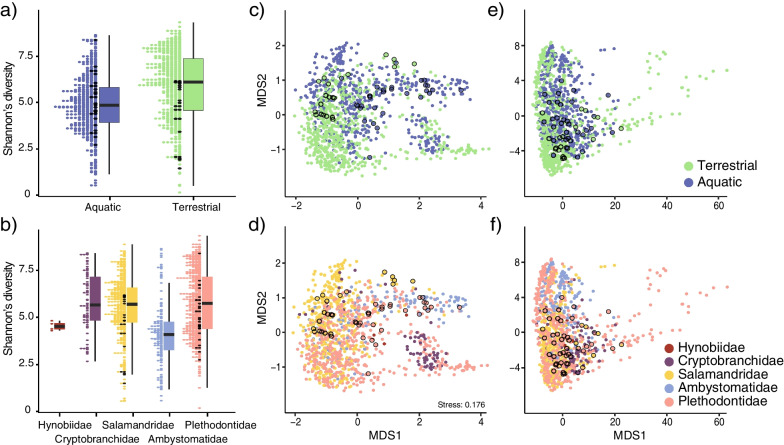
Influence of host habitat and taxonomy on the diversity of the salamander skin bacterial communities. **a**–**b**, distribution of bacterial alpha diversity (Shannon Diversity Index) by salamander habitat (**a**) and family (**b**). Black circles correspond to samples from captive host species. **c**–**f** non-metric multidimensional scaling (nMDS) of beta diversity estimated using weighted Unifrac (wUF) (**c, d**) and Unweighted Unifrac (uwUF) (**e, f**) distances. Colours are indicative of the corresponding classifications. Circles with black outline correspond to samples from captive host species

To further explore differences in bacterial diversity across salamander hosts we calculated bacterial alpha (Shannon Diversity Index) and beta diversity, using weighted (wUF) and unweighted Unifrac (uwUF) distances, and compared these estimates across host families and habitats (Fig. 3). Specifically, samples from aquatic habitats had lower alpha diversity relative to those from terrestrial habitats (median Shannon Diversity: aquatic = 4.85, terrestrial = 6.10; Wilcoxon’s W = 109,488, *df* = 1, *p* < 0.01; Fig. 3a). Alpha diversity also differed among salamander families (Kruskall–Wallis x^2^ = 102.67, *df* = 4, *p* < 0.01; Fig. 3b) and family Ambystomatidae had lower values than Plethodontidae (pairwise W = 19,827, *df* = 1, p-adjusted < 0.01), Salamandridae (pairwise W = 47,280, *df* = 1, p-adjusted < 0.01), and Cryptobranchidae (pairwise W = 8112, *df* = 1, p-adjusted < 0.01). Variation in bacterial alpha diversity was also found among salamander species (Kruskall–Wallis x^2^ = 313.48, *df* = 43, *p* < 0.01) (Additional file 1: Fig. S3). For bacterial beta diversity, the PERMANOVAS (Permutational multivariate analysis of variance) showed dissimilarities in skin bacterial composition and structure between host sampling habitats (uwUF: F = 5.71, *df* = 1, *p* = 0.001; wUF: F = 21.69, *df* = 1, *p* = 0.001) and among host families (uwUF: F = 40.5, *df* = 4, *p* = 0.001; wUF: F = 24.11, *df* = 4, *p* = 0.001), but an overall low proportion of variance was explained by either of these factors (all R^2^ < 0.15) (Fig. 3c–f).

### Climate influences skin bacterial diversity

We fitted a linear mixed model to assess the influence of climatic variables on alpha diversity of the salamander skin bacteria, while accounting for the effects of host habitat and taxonomy. Our model included host sampling habitat, host family, seven bioclimatic variables, elevation, and two monthly variables as fixed effects, while including datasets as random effects (Additional file 1: Table S1). This model showed that bacterial alpha diversity varied the most as a function of salamander habitat and family, yet climatic variables also had a non-negligible influence on alpha diversity (Additional file 1: Fig. S4). The fixed effects of this model accounted for 23.6% of the observed variance (marginal R^2^); when the random effects were considered (conditional R^2^) the model accounted for 33.1% of the variance.

In the multivariate context, salamanders sampled from terrestrial habitats showed higher levels of bacterial alpha diversity relative to those sampled from aquatic habitats (the reference level), whereas those belonging to families Cryptobranchidae, Plethodontidae, and Salamandridae exhibited higher levels of alpha diversity relative to those from family Ambystomatidae (the reference level) (Additional file 1: Fig. S4). The model included climatic variables associated with temperature and precipitation and elevation, but only one bioclimatic variable (precipitation of the driest quarter, bio17) showed a significant negative effect on bacterial alpha diversity (Additional file 1: Fig. S4). The model indicated that alpha diversity was lower in samples taken from localities with higher dry season precipitation (while controlling for all other factors), indicating that samples from localities with more pronounced ‘dry’ seasons tend to have more diverse bacterial assemblages.

To disentangle the relative contributions of climatic and host factors on bacterial beta diversity we performed a distance-based redundancy analysis (dbRDA) using the wUF and uwUF dissimilarity matrices; we fitted models using both climatic and host factors. Our models for both wUF and uwUF included the effects of host sampling habitat and family, nine bioclimatic variables, and two monthly variables (Additional file 1: Tables S2–S3). For wUF, we retrieved 12 statistically significant canonical axes (*p* value < 0.05) that collectively explained 25.26% of the observed variance in beta diversity across samples; for uwUF we retrieved 16 statistically significant axes explaining 14.45% of the variance. Overall, our models showed that climatic variables had a largest influence relative to host sampling habitat and family on skin bacterial composition (uwUF) and structure (wUF). A PERMANOVA over each variable showed bio2 (mean diurnal range), precipitation, and bio18 (precipitation of the warmest quarter) had the three largest effect-sizes (*p* = 0.001; *df* = 1) on both the wUF and uwUF matrices (Additional file 1: Table S2–S3). Host sampling habitat had a significant (*p* = 0.001; *df* = 4), but smaller effect-size on both wUF and uwUF distances.

### Salamander host phylogeny is correlated with skin bacterial community structure

**Fig. 4:**
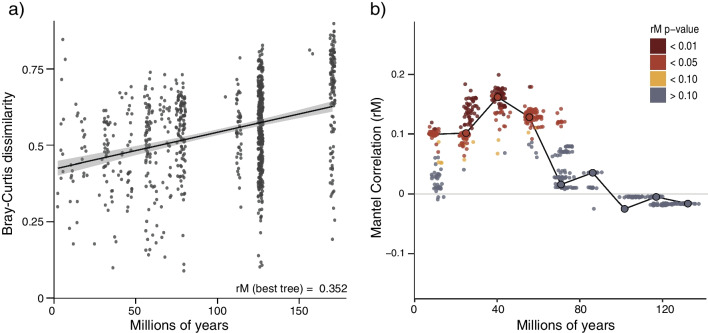
Association between salamander phylogenetic distances and skin bacterial community dissimilarity. **a** Bacterial dissimilarity (Y-axis) at the order level as a function of host species evolutionary distances (X-axis) estimated by fossil-based molecular dating of the best-scoring ML tree of extant salamanders. The solid black line represents the slope estimated with a Mantel test between matrices. **b** Correlogram showing the variation in the Mantel correlation coefficients as a function of host species evolutionary distances (in millions of years). Open circles connected by a solid black line represent the correlations estimated with the best-scoring ML tree. Solid circles represent the correlations estimated with evolutionary distances using the fossil-based molecular dating of the bootstrap trees. Colours are indicative of the corresponding p-values of correlations

Based on mitochondrial and nuclear loci, we reconstructed a Maximum Likelihood phylogenetic tree for 580 species of Caudata (~ 82% of extant salamander species) and performed fossil-based molecular dating (Additional file 1: Fig. S1). We assessed the influence of the salamander host phylogeny on bacterial beta diversity by employing Mantel and partial Mantel tests using pairwise patristic distances (here in time units) and bacterial Bray–Curtis dissimilarities at different levels of bacterial taxonomy. To account for topological and branch-length uncertainty in the salamander phylogeny, we estimated Mantel correlations using a sample of 100 bootstrap trees plus the best scoring ML tree (n = 101), resulting in median rM and rMp estimates ranging from 0.04 to 0.35 and 0.01–0.26, respectively, across bacterial taxonomic levels (Additional file 1: Fig. S5). Overall, the Mantel correlation tests consistently revealed a significant positive phylogenetic signal in skin bacterial structure at the bacterial order and family levels (Fig. 4; Additional file 1: Fig. S6; Additional file 1: Table S4), where the dissimilarity of skin bacterial assemblages increased as evolutionary distances increased among host species (Fig. 4a). The partial Mantel tests consistently retrieved significant positive correlations, after controlling for climatic distances among host species, only for the bacterial order level (Additional file 1: Fig. S5).

To test the evolutionary scale at which positive phylogenetic signals were occurring, we estimated Mantel correlograms to assess how rM varied at different temporal scales across the salamander phylogeny. While accounting for uncertainty in the host phylogeny, we found a positive phylogenetic signal of bacterial order composition (Fig. 4b), but only within the first four distance classes that are roughly equivalent to the last 50 million years of salamander evolution (Fig. 4b). This pattern was also observed when using bacterial dissimilarity matrices at the family level (Additional file 1: Fig. S6).

## Discussion

Here, we assessed the relative contributions of host habitat, climate, and host phylogenetic relationships to the diversity and structure of skin bacterial communities in salamanders. In agreement with previous studies on amphibian skin microbiota, we found that host sampling habitat (terrestrial/aquatic) [13, 30], precipitation, and seasonality play a major role in shaping the diversity of salamander skin bacterial communities [9, 14, 15, 22]. We also inferred that host phylogenetic relationships have an important effect in shaping these bacterial communities [22], which contrasts with previous studies in which phylogenetic effects were minor [9, 13, 26].

A recent study across a wide diversity of animal-associated microbiomes showed that bioclimatic variables related to temperature and precipitation were relevant in shaping host-associated external microbiomes, in contrast with internal microbiomes which are mainly influenced by host diet and phylogeny [11]. Specifically for amphibians, two studies on anurans at continental to global scales explored the relative contributions of distinct biotic and abiotic factors and found evidence that skin bacterial diversity is mostly influenced by long-term temperature and precipitation averages [9, 14]. Our findings agree with these results in revealing an effect of average climate regimes (specifically precipitation seasonality [9]) on the salamander skin bacterial diversity. However, relying on long-term climate averages (e.g., yearly bioclimatic variables from WorldClim [31]) leads to loss of information on local, year-to-year variations in climate; in this case, samples can share the same climate averages but differ in their levels of bacterial diversity due to short-scale temporal variation. Indeed, by incorporating monthly climatic variables into our analyses, we found that precipitation at time of sampling (month) had a significant and positive effect on bacterial beta diversity. This agrees with observations of significant variation in bacterial communities across long- and short-term time scales [8, 13, 15]. We hypothesise that long-term seasonal effects may explain higher bacterial alpha diversity in salamanders’ skin due to increased temporal turnover in community composition [9]. In addition, short-term increases in precipitation may result in higher bacterial turnover due to increased interchange of bacteria across multiple sources facilitated by rain and water movement across the ecosystem [32]. To further explore the temporal and spatial dynamics of amphibian skin microbiomes, researchers should include more precise spatial and temporal data on climate and other environmental factors (*e.g.*, water pH, salinity), and more detailed information on host’s life history traits and behaviour at the population level.

We found that local-scale host sampling habitat (e.g., terrestrial, aquatic) had a major influence on skin bacterial alpha and beta diversity. Environmental bacteria are considered one of the main sources of microbial diversity for amphibian skin microbiomes [25, 26, 33, 34], and evidence has shown that host habitat is one of the major drivers of anuran and caudate skin microbial diversity [12, 26, 30, 35]. Our results showed differences between individual salamanders sampled from aquatic and terrestrial habitats and that specific bacterial orders differed in relative abundances between these habitats. The sampling habitats we included do not reflect the entire set of habitats explored by salamander species and only refer to the habitat where individuals were found. Therefore, the bacterial communities described here are likely a subset of the species’ entire bacterial diversity. We also identified a set of bacterial taxa shared among all sampled salamander species, yet only a small proportion of salamander species have been sampled and several salamander families remain unsampled (Additional file 1: Fig. S1). These results should be taken with caution because varying sample effort across salamander hosts likely impacts estimation of bacterial prevalence across samples. Furthermore, the data we gathered revealed that most studies on salamander microbiomes are focused on single host species from either aquatic or terrestrial habitats; out of 21 studies, only four included samples from both habitats, whereas only three included samples from different families. This complicates teasing apart the influence of study design (*e.g.*, differences in sampling or sequencing among studies [36]) from that of biological factors (*e.g.*, habitat or family).

Our results showed that host habitat and family were confounded and some bacterial taxa appeared enriched simultaneously by both factors. Thus, some of the differences in bacterial relative abundances we see across habitats may be related to host phylogenetic history. Indeed, our analyses show that the host family, independent of habitat, is an important factor influencing alpha and beta diversity of skin bacterial communities in salamanders. These results agree with previous inference on a phylogenetic effect when comparing skin bacterial communities between different host orders [20] or genera within families [22, 27]. However, in other cases the effect of habitat/environment has been stronger in host species within the same genera [26] or genera within the same family [37]. Most of these analyses do not use direct measures of phylogenetic distance among species (e.g., divergence times or branch lengths) and instead rely on comparisons among different taxonomic entities (e.g., genera or families).

To tackle the effect of host phylogeny on skin bacterial diversity we constructed a dated salamander phylogeny and directly used branch-length distances among host species (in millions of years). By doing this, we found a significant role of host phylogenetic relationships in shaping skin bacterial composition, even after controlling for climatic differences among host ranges. More specifically, we found positive significant correlations between bacterial community distances (Bray–Curtis) and host phylogenetic distances, where similarity in salamander skin bacterial communities increases with decreasing host phylogenetic distance. These correlations are robust to topological and divergence time uncertainty of the salamander phylogeny. In other words, we uncovered a general tendency where skin bacterial communities of closely related host species resemble each other more than those of host species drawn at random from the same tree. Recent meta-analyses spanning several amphibian families (mainly anurans) have found significant but weaker effects of host phylogeny (relative to other factors) using topological congruence analysis and other proxies of host phylogeny (i.e., nMDS of patristic distances) [9, 11, 12, 14]. In these cases, the weaker phylogenetic signal probably stems from loss of statistical power because distances in microbiota compositions based on dendrograms or nMDS and raw (true) distances are moderately to poorly correlated [4].

Furthermore, based on the results of previous studies [20, 26, 27, 37] we believe that the scale at which the host-phylogenetic effect is being analysed might explain some of the discrepancies found on the strength of phylogenetic effects. Interestingly, we observed that the phylogenetic effect on salamander skin bacteria was stronger at intermediate levels of host divergence, even after controlling for climatic distances among host species, roughly corresponding to the last 50 million years of salamander evolution, and that deeper salamander divergences do not correlate to skin bacterial differentiation. We also observed that the phylogenetic signal was significant, albeit with varying strength, when using bacterial dissimilarities at different taxonomic levels (i.e.*,* class, order, family). Although the phylogenetic signal decreased at higher taxonomic ranks, the current 16S data do not allow for a robust test at higher bacterial taxonomic ranks due to uncertainty in taxonomic assignments (*e.g.*, genera).

Host-mediated environmental filtering (through traits unaccounted for in the analyses) may be playing a substantial role in determining skin bacterial composition in salamanders. Phylogenetic signals can be produced by host-mediated ecological filtering, in which ﻿host traits selectively filter microbes from the environment [4–6]. Internal microbiomes (e.g., gut) have been shown to have strong phylogenetic signal [4, 6, 38, 39], whereas superficial microbiomes (e.g., skin) show weaker phylogenetic signals, specifically in amphibians [9]. This is thought to be the result of the latter being more prone to the effects of exogenous factors converging across the host phylogeny (e.g., climatic niche preferences). The strength of the phylogenetic signal would depend on the degree of phylogenetic correlation of the specific traits involved in ecological filtering of microbes. In our case, we assessed the weight of climate preferences of hosts in explaining the phylogenetic signal in skin bacterial communities and found that climatic distances explain some of the variance in beta diversity across hosts; however, most of the phylogenetic signal remains unaccounted by these climate factors.

Overall, skin bacterial similarity in salamanders appears to be driven by recent host (and bacterial) evolution [4, 39]. The evidence of phylogenetic signal across multiple levels of host divergences does not support an overarching effect of ecological filtering through environmental host preferences (climate and habitat), at least at the level of salamander families. However, our findings do not preclude an important role of host-mediated ecological filtering of skin microbes occurring at lower taxonomic ranks (e.g., between salamander genera or closely related species). Here, we argue that phylogenetic signal associated with variation in specific putative traits (e.g., genetic diversity, major histocompatibility complex, antimicrobial peptides) may be important to explain differences in skin bacterial composition, but that these putative traits are probably associated with the evolutionary history of salamander hosts [6].

## Materials and methods

### 16S rRNA amplicon sequence data

We gathered available published data on skin bacterial communities from salamander species (order Caudata) (last updated, December 2022) from 20 studies [13, 15, 17, 20, 25, 27, 30, 40–52]; these include data deposited in the National Center for Biotechnology Information (NCBI), the European Bioinformatics Institute (EBI), the Dryad repository, and additional data obtained through peer-to-peer data requests. We performed an extensive search using the SRA Run Selector tool from NCBI (https://0-www-ncbi-nlm-nih-gov.brum.beds.ac.uk/Traces/study/) to select studies with publicly available 16S rRNA sequence data generated with the Illumina platform. We downloaded the SRA sequences and associated metadata from Entrez search results. Most studies had sequence data for the V3–V4 and V4 ribosomal regions, but a couple of studies sequenced the ribosomal regions V2 and V3 (Additional file 2). Metadata obtained from each of the studies included: locality (latitude and longitude), sampling habitat (terrestrial/aquatic), sequencing primers, sequencing technology, collection date, and sample origin (wild/captive). Sampling habitat was determined based on information obtained from the metadata and methods originally provided in the published datasets/papers; in several cases we consulted directly with authors about sample provenance. This variable refers to the place and time where the animals were found and sampled and does not describe the life history trait of the salamander species. We believe that this approach is more suitable to study bacterial assemblages because it has been shown that animals of the same species sampled in different habitats show marked differences in bacterial composition (e.g., Sabino-Pinto et al. [30]).

The publicly available data and metadata we used included 16S rRNA amplicon sequences for 1031 samples from 37 salamander species. We added new bacterial data for seven Mexican salamander species generated by our working group. Specifically, we included 111 samples for six species of Plethodontidae (*Aquiloeurycea cafetalera*, *Chiropterotriton nubilus*, *Parvimolge townsendi*, *Pseudoeurycea granitum*, *Pseudoeurycea lynchi, Pseudoeurycea nigromaculata*) and 22 samples of *Ambystoma mexicanum* (Ambystomatidae). These species were sampled from wild populations except for *A. mexicanum,* which were sampled from simulated outdoor environments (mesocosms). For these five species, we obtained samples after rinsing the skin with 25 ml of sterile water to eliminate transient microorganisms and then swabbing the skin with a sterile cotton swab. We extracted total genomic DNA from each swab using a Qiagen DNeasy Blood and Tissue kit (Qiagen, Germantown, USA) and amplified the V4 region using barcoded primers (515F–806R). Single-end amplicons were sequenced using an Illumina MiSeq 300 platform at the Dana Farber Cancer Institute. We also extracted and sequenced negative controls (dummy swabs), but these did not amplify during 16S library construction and thus were not included in subsequent sequencing.

The complete dataset includes the following samples taken from captive animals (Additional file 2): *Echinotriton andersonii* (22 samples), and *Eurycea waterlooensis* (28 samples). For *E. andersonii and E. waterlooensis,* captive individuals were taken from indoor environments that are not representative of their native habitat. All samples from captive individuals were included in the estimation of diversity metrics and bacterial relative abundances.The samples for *A. mexicanum* were taken from animals under simulated outdoor environments (mesocosms) that are located within the species’ former native range (Xochimilco Lake). These are exposed to the same climate conditions as the original natural habitat and use water sourced directly from the lake. Considering the above, samples for *A. mexicanum* were included in all analyses including linear mixed models and Mantel tests.

In sum, the 16S amplicon sequence data comprised 1,164 samples and contained a total of 2,677,200 reads, which were processed using semi-automated pipelines in QIIME2 version 2021.2.0 [53]. Prior to importing the sequence data into QIIME2, we aligned and assembled forward and reverse reads using *﻿Paired-End reAd mergeR* [54] (PEAR) and discarded sequence reads with a quality score > 20 and a length < 100bp using *Trimommatic* [55]*.* We then processed the filtered data using QIIME2. All samples were rarefied to 2,300 reads per sample. Sequences were grouped by the study of origin and processed using DADA2 [56]. To merge sequence reads from different 16S regions, we used the plugins ‘feature-table merge’ and ‘fragment-insertion’ implemented with the SILVA database tree in QIIME2 [53]. This allowed estimation of alpha and beta diversity indices at the ASV level.

*Climate and elevation data.* We obtained bioclimatic data for each sampled locality using the corresponding geographic coordinates (latitude and longitude) provided in the original studies. We extracted information for the 19 bioclimatic variables from WorldClim 2.1 [31] at a 30 s (~ 1 km^2^) spatial resolution; these data represent climate averages over the period 1970–2000 (Additional file 2). In addition, we used the reported collection date (month and year) at each sampling locality to obtain historical climate data from the monthly series available in WorldClim 2.1 [31]. These climate series include monthly data for precipitation and temperature (minimum and maximum) over the period 1960–2018. For each sample, we used the GPS coordinates to extract data for the corresponding month/year with a spatial resolution of 30 s; for 193 samples taken in the period 2019–2021, we used the latest data available from 2018. We extracted elevation data for each locality from the GTOPO30 global digital elevation model (US Geological Service’ Earth Resources Observation and Science Center) with a spatial resolution of 30 arc-seconds. Data extraction was performed using the *raster* [57] and *sp* [58, 59] packages in R [60].

### Salamander phylogeny

To assess the influence of host phylogeny, we constructed a species-level dated phylogeny for salamanders and estimated evolutionary distances among species. Briefly, we assembled a molecular sequence alignment for Caudata from NCBI’s GenBank vertebrate database (last updated May, 2020) using the semi-automated pipeline PyPHLAWD [61]; this automatic pipeline retrieves molecular data from GenBank, generates ‘clusters’ of likely ortholog sequences using the Basic Local Alignment Search Tool [62], and aligns each cluster with the Multiple Alignment using Fast Fourier Transform (MAFFT) algorithm [63]. We queried for ‘Caudata’ sequences longer than 400 bp, with a minimum sequence identity of 0.2, and a minimum coverage of 0.65. We complemented the molecular matrix with sequence data for *Ambystoma marvotium*, *A. tigrinum*, and an unnamed species (*Pseudoeurycea sp*) with available 16S sequence data, which were aligned using Clustal2.0 [64] and AliView [65]. The resulting alignment included both nuclear and mitochondrial markers, with a total length of 224,266 bp for 580 living species of salamanders.

We estimated a maximum likelihood (ML) phylogeny with RAxML v.8 [66] using the GTRCAT model, 1000 bootstrap replicates, and substitution parameters estimated for each partition independently. We constructed an initial ML tree to check for ‘rogue taxa’ and evaluate the overall accuracy of the estimated tree topology. After this initial check, we constructed a final ML tree where we conservatively applied several constraints on the topology of the tree and used three species of *Xenopus* (Anura) as the outgroup. Overall, the ML tree showed high support values (> 0.85) for relationships at the genus level and above, but lower support for relationships at the species level (Additional file 3). We compiled a set of 38 fossil specimens for Caudata to perform fossil-based molecular dating (Additional file 4); in sum these fossils calibrate 18 distinct nodes in the salamander phylogeny. The relations of fossils to extant species were based on the original assignments of fossils and their phylogenetic relationships were vetted against the proposal by Marjanovic and Laurin [67]. We performed the fossil-based molecular dating of the ML tree with a Penalized Likelihood (PL) approach as implemented in *treePL* [68], with a smoothing parameter of 0.00001 that was determined through cross-validation; we dated the best-scoring tree and 100 bootstrap trees to account for uncertainty in divergence time estimates across the phylogeny (Additional files 5 and 6). For all dating analyses, we set an age constraint for the stem and crown nodes of Caudata of 227–280 Mya and 166.1–280 Mya, respectively.

### Bacterial diversity analyses

We used QIIME2 [53] to assign bacterial taxonomy to ASVs using the Ribosomal Database Project [69] and estimate the relative abundance of bacterial taxa across all samples. We employed the core-metrics-phylogenetic pipeline in QIIME2 to estimate alpha and beta diversity using the estimates of the relative abundance of ASVs across samples. We estimated bacterial alpha diversity using Shannon Diversity index (Additional file 2) and estimated bacterial beta diversity using the phylogenetic-based weighted (wUF) and unweighted (uwUF) Unifrac dissimilarity indices (Additional file 7). We explored differences in microbial alpha diversity among salamander sampling habitats and families using a Wilcoxon test and a Kruskal–Wallis test, respectively; we also performed pairwise Wilcoxon tests to assess differences among families using a Bonferroni correction for multiple comparisons. We explored differences in beta diversity among salamander habitats and families by performing a non-metric multidimensional scaling (nMDS) on the wUF and uwUF matrices, followed by a Permutational Analysis of Variance (PERMANOVA) [70]. We did not attempt to use stratification by study ID in alpha and beta analyses because most studies were performed on single species from one habitat type. A stratified permutation would not be appropriate because permutations would be limited to within studies [70], leading to permutation of samples with the same grouping variables. All statistical tests were performed using the *vegan* [71] package in R [60].

We searched for shared bacterial orders and families among all salamander species using their relative abundances; for this we used the taxonomy of ASVs at level four and five of the Ribosomal Database Project, which in general coincide with bacterial orders and families. However, there are exceptions, for which additional subclass levels are included in the taxonomic annotation and thus levels four and five correspond to subclass and order, respectively. Thus, we manually edited the corresponding taxonomy of some ASVs to match levels 4 and 5 with orders and families, respectively. We then estimated the prevalence of shared bacterial taxa within each host species; for each bacterial taxon and host species, prevalence was estimated as the percentage of host samples where the presence of a bacterial taxa was detected. Finally, we assessed whether particular bacterial taxa could discriminate among samples from different salamander habitats or families by employing a Linear discriminant Effect Size analyses (LEfSe) [72] using habitat and family as response variables, separately. The LEfSe analyses were performed using the relative abundances tables at the bacterial-order level and only those bacterial taxa with an LDA scores > 2.0 were considered as informative [35, 72–74]. For the LEfSE analysis using host families we employed a ‘strict’ strategy [72] to identify differentially abundant bacterial taxa; here the abundance profile of a feature (taxa) has to be significantly different among all classes tested (families).

### Drivers of bacterial alpha diversity

To assess the influence of different factors on bacterial phylogenetic alpha diversity (log-transformed Shannon Diversity Index), we fitted a linear mixed model that included the fixed effects of host sampling habitat, host family, climatic variables and elevation, while controlling for the possible random effects on the intercept across studies; these random effects are aimed to encapsulate differences in levels of alpha diversity among studies due to sampling and sequencing techniques. We implemented a two-step approach to select the least-correlated bioclimatic variables that remained as strong predictors of bacterial alpha diversity: (1) a stepwise forward and backward regression that uses the Akaike Information Criteria (AIC) to select bioclimatic variables with significant effects on alpha diversity; (2) pairwise Pearson correlations among selected variables to identify and discard those with a pairwise correlation higher than r > 0.7. We used the selected variables, together with salamander sampling habitat and family, to fit a linear mixed model using the *lme4* [75] package in R [60]. The resulting model was further simplified by estimating variance inflation factors (VIFs) of all variables using the *performance* [76] package in R [60]. We identified and discarded variables with a VIF > 10 and fitted a new simplified linear mixed model; the fitted model takes the form (see Additional file 2 for variable names):

$$\begin{gathered} {\text{Shannon Diversity}}\sim {\text{pre}} + {\text{tm}}\_{\text{max}} + {\text{elevation}} + {\text{bio2}} + {\text{bio6}} + {\text{bio8}} + {\text{bio1}}0 + {\text{bio17}} \hfill \\ + {\text{bio18}} + {\text{bio19}} + {\text{Habitat}} + {\text{Family}} + \left( {{1 }|{\text{ Dataset}}} \right) \hfill \\ \end{gathered}$$

### Drivers of bacterial beta diversity

To determine the major factors influencing bacterial beta diversity we employed a distance-based redundancy analysis (dbRDA) [77] to evaluate the influence of host family, host sampling habitat, climatic variables, and elevation on bacterial beta diversity; we used the wUF and uwUF dissimilarity matrices as the response variables, separately. Briefly, the dbRDA performs classical multidimensional scaling on a dissimilarity matrix and then conducts a redundancy analysis using the ordination scores to examine how much variation is explained by a given set of explanatory variables [74]. Prior to the analyses, we z-scored the climatic variables and performed variable selection as described above for the linear mixed model. We used the selected variables, together with salamander sampling habitat and family, to perform dbRDA using the *vegan* package [71] in R [60] and employed a permutational approach to test for significance of the effect of individual predictor variables. After identifying and discarding variables with a VIF > 10, the final fitted model takes the form (see Additional file 2 for variable names):

$${\text{Beta diversity}}\sim {\text{pre}} + {\text{tm}}\_{\text{max}} + {\text{bio2}} + {\text{bio8}} + {\text{bio17}} + {\text{bio19}} + {\text{Habitat}} + {\text{Family}}$$

### Host phylogenetic effect

We used the dated salamander phylogeny to explore the correlation between host phylogenetic distances and bacterial community distances using the Bray–Curtis dissimilarity index. More specifically, we used Mantel and partial Mantel tests to assess the strength of the correlation between host phylogenetic distances and microbiome dissimilarity, while controlling for climatic distances among host species; in other words, our Mantel tests can be formulated as assessments of whether microbiome distances are structured in ‘phylogenetic space’. For this, we obtained a Bray–Curtis dissimilarity matrix by averaging the ASV relative abundances across samples for each salamander species and then used the ASV assigned taxonomy to estimate the relative abundances of bacterial taxa at different taxonomic ranks (Additional file 8). We estimated host phylogenetic distances as pairwise patristic distances among salamander species, which were measured in millions of years since the most recent common ancestor for each pair of species. We estimated evolutionary distances for each of the dated phylogenetic trees obtained with *treePL* (bootstrap trees and best scoring tree, n = 101) using the *adephylo*^78^ package in R [60]. We estimated climatic distances between species using the climatic data extracted for every sampled locality in our database and performed a Principal Component Analysis (PCA) of the climatic variables using the *ade4*^79^ package in R [60]; we summarized the scores for the principal components for each salamander species and estimated the pairwise Euclidean distances between all pairs of salamander species to obtain a climatic dissimilarity matrix.

Finally, we employed Mantel correlograms to evaluate the evolutionary scale at which correlations between host phylogenetic and bacterial community dissimilarities are occurring. The correlogram depicts the variation in the Mantel correlation as a function of phylogenetic distance classes, which are estimated directly from the data; we corrected for multiple comparisons in the correlograms using the false discovery rate (fdr). We used the *mpmcorrelogram*^80^ package in R [60] to estimate Mantel correlograms while controlling for climatic distances (partial correlograms). For all tests, we evaluated significance by performing 999 permutations.

### Supplementary Information

**Additional file 1.** Supplementary tables and figures.

**Additional file 2.** 16S data summary and sample metadata.

**Additional file 3.** Salamander Maximum Likelihood phylogeny.

**Additional file 4.** Fossil calibration list.

**Additional file 5.** Salamander time-calibrated best-scoring phylogeny.

**Additional file 6.** Salamander ime-calibrated boostrap phylogenies.

**Additional file 7.** Unifrac dissimilarity matrices for 16S samples.

**Additional file 8.** Bray-Curtis dissimilarity matrices at different bacterial taxonomic ranks.

**Additional file 9.** Relative abundances of bacterial orders across host species.

**Additional file 10.** Relative abundances of bacterial families across host species.

**Additional file 11.** Results of the LefSe for bacterial orders and families between host habitats.

**Additional file 12.** Results of the LefSe for bacterial orders and families among host families.

## Data Availability

The code and data supporting the results in the main text are available at https://github.com/spiritu-santi/salamanders and https://github.com/PacoMax/Microbiota_V4. Metadata for each sample is provided as supplementary data. Raw sequence data are publicly available (Bioprojects are listed in Additional file 1). Newly generated data can be found as Bioprojects PRJNA924965 and PRJNA926363.
