# Supplementary material for: Host phylogeny and environment shape the diversity of salamander skin bacterial communities
Source: Anim Microbiome. 2023 Oct 13;5:52. doi: 10.1186/s42523-023-00271-7 (PMC10571319; doi:10.1186/s42523-023-00271-7)
Supplement: Supplementary file 1 — Additional file 1. Supplementary tables and figures. [file 42523_2023_271_MOESM1_ESM.docx]

**Supplementary Tables and Figures**

**Host phylogeny and environment shape the diversity of the salamander bacterial communities**

Ramírez-Barahona S^1,†^, González-Serrano FM^2,†^, Martínez-Ugalde E^2^, Soto-Pozos A^2,3^, Parra-Olea G^3^, Rebollar EA^2,^*

**Supplementary table S1.** Fixed and random effects of climatic and host factors on alpha diversity estimated using Linear Mixed Models. The reference level for salamander family is Ambystomatidae and the reference level for host habitat is Aquatic. Terms (except the intercept and the random effects) are arranged based on the absolute value of the model estimates (see supplementary data 1 for variable names).

| Type of effect | Term | Estimate | Std error | t statistic | Lower CI | Upper CI |
| --- | --- | --- | --- | --- | --- | --- |
| fixed | Intercept | -1.17 | 0.24 | -4.77 | -1.65 | -0.69 |
| fixed | Cryptobranchidae | 1.50 | 0.38 | 3.91 | 0.75 | 2.25 |
| fixed | Plethodontidae | 1.14 | 0.29 | 3.87 | 0.56 | 1.72 |
| fixed | Salamandridae | 1.09 | 0.30 | 3.65 | 0.51 | 1.68 |
| fixed | Habitat | 0.43 | 0.11 | 4.06 | 0.23 | 0.64 |
| fixed | Hynobiidae | -0.41 | 0.64 | -0.63 | -1.67 | 0.85 |
| fixed | bio17 | -0.27 | 0.11 | -2.46 | -0.49 | -0.06 |
| fixed | elevation | -0.18 | 0.18 | -1.01 | -0.53 | 0.17 |
| fixed | bio10 | -0.14 | 0.14 | -0.97 | -0.42 | 0.14 |
| fixed | bio18 | 0.10 | 0.09 | 1.14 | -0.07 | 0.27 |
| fixed | bio19 | -0.08 | 0.08 | -0.99 | -0.25 | 0.08 |
| fixed | bio6 | -0.06 | 0.10 | -0.60 | -0.24 | 0.13 |
| fixed | bio8 | -0.06 | 0.07 | -0.87 | -0.18 | 0.07 |
| fixed | pre | 0.03 | 0.05 | 0.67 | -0.06 | 0.12 |
| fixed | tm_max | 0.01 | 0.05 | 0.30 | -0.08 | 0.10 |
| fixed | bio2 | 0.01 | 0.10 | 0.08 | -0.19 | 0.20 |
| random | Dataset | 0.32 |  |  |  |  |
| random | Residuals | 0.85 |  |  |  |  |

**Supplementary table S2.** Effect-sizes obtained with the distance based redundancy analyses (dbRDA) of microbial beta diversity as a function of climate and host factors using the weighted Unifrac (wUF) distance matrix. Terms are arranged based on their absolute the estimated pseudo-F values (see supplementary data 1 for variable names).

|  | Degrees of freedom | Sum of squares | Pseudo-F | p-value |
| --- | --- | --- | --- | --- |
| bio2 | 1 | 46.60 | 52.8081 | 0.001 |
| pre | 1 | 30.71 | 34.8036 | 0.001 |
| bio18 | 1 | 27.77 | 31.462 | 0.001 |
| bio4 | 1 | 26.99 | 30.5862 | 0.001 |
| bio8 | 1 | 25.76 | 29.1863 | 0.001 |
| Family | 4 | 98.03 | 27.7692 | 0.001 |
| bio17 | 1 | 23.62 | 26.7645 | 0.001 |
| bio1 | 1 | 15.55 | 17.6194 | 0.001 |
| Habitat | 1 | 14.50 | 16.4324 | 0.001 |
| tm_max | 1 | 9.54 | 10.8112 | 0.001 |
| bio19 | 1 | 8.66 | 9.8128 | 0.001 |
| Residual | 1099 | 969.89 |  |  |

**Supplementary table S3.** Effect-sizes obtained with the distance based redundancy analyses (dbRDA) of microbial beta diversity as a function of climate and host factors using the unweighted Unifrac (uwUF) distance matrix. Terms are arranged based on their absolute the estimated pseudo-F values (see supplementary data 1 for variable names).

|  | Degrees of freedom | Sum of squares | Pseudo-F | p-value |
| --- | --- | --- | --- | --- |
| bio2 | 1 | 8.80 | 28.35 | 0.001 |
| bio18 | 1 | 5.50 | 17.72 | 0.001 |
| pre | 1 | 4.48 | 14.44 | 0.001 |
| bio17 | 1 | 4.16 | 13.39 | 0.001 |
| bio4 | 1 | 4.01 | 12.93 | 0.001 |
| bio8 | 1 | 3.69 | 11.88 | 0.001 |
| Habitat | 1 | 3.57 | 11.50 | 0.001 |
| Family | 4 | 14.14 | 11.39 | 0.001 |
| bio1 | 1 | 2.84 | 9.16 | 0.001 |
| tm_max | 1 | 2.25 | 7.24 | 0.001 |
| bio19 | 1 | 2.04 | 6.57 | 0.001 |
| Residual | 1099 | 341.22 |  |  |

**Supplementary table S4.** Mantel (rM) and partial mantel (prM) correlations between bacterial dissimilarity and host phylogenetic distances at different levels of bacterial taxonomy. Point estimates are given for the best Maximum Likelihood tree (ML) and summary statistics are given for estimates across the 100 bootstrap trees.

| Bacterial taxonomy | rM median | rM minimum | rM maximum | prM median | prM minimum | prM maximum | rM  ML tree | prM  ML tree |
| --- | --- | --- | --- | --- | --- | --- | --- | --- |
| Phylum | 0.04 | 0.02 | 0.06 | 0.01 | -0.01 | 0.03 | 0.04 | 0.01 |
| Class | 0.28 | 0.22 | 0.30 | 0.19 | 0.13 | 0.22 | 0.28 | 0.19 |
| Order | 0.35 | 0.27 | 0.38 | 0.26 | 0.18 | 0.29 | 0.35 | 0.26 |
| Family | 0.30 | 0.23 | 0.33 | 0.19 | 0.13 | 0.22 | 0.30 | 0.19 |
| Genus | 0.19 | 0.15 | 0.22 | 0.10 | 0.07 | 0.13 | 0.19 | 0.10 |
| ASV | 0.21 | 0.19 | 0.23 | 0.07 | 0.05 | 0.09 | 0.22 | 0.07 |

**Supplementary figures**

**
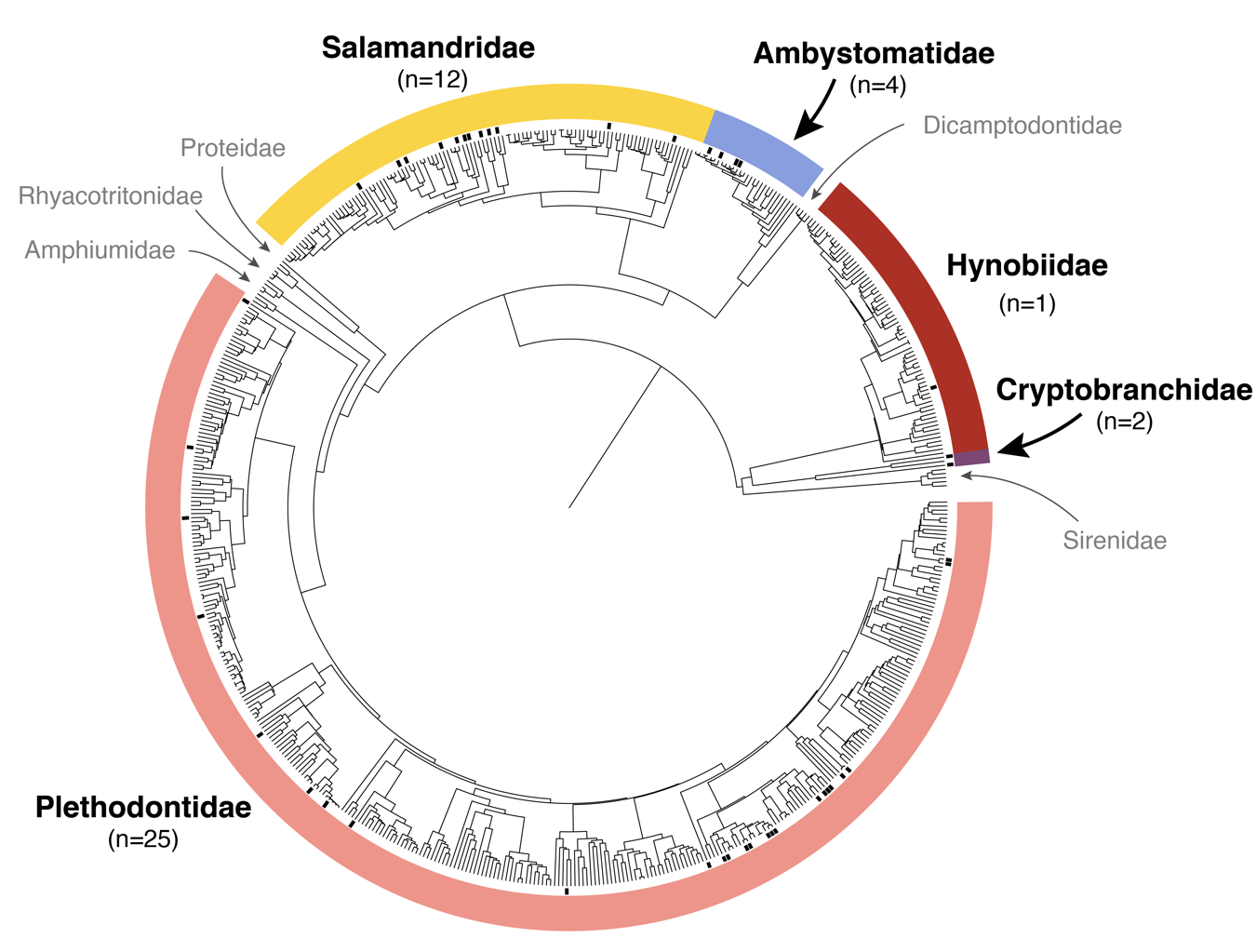
**

**Supplementary fig. S1. Species-level phylogeny for salamanders.** Black squares at the tips of the phylogeny depict the salamander species with available skin microbiome data. Outer circle depicts the ten recognized salamander families and the names in bold represent families with available skin microbiome data (n = number of species with data).

**
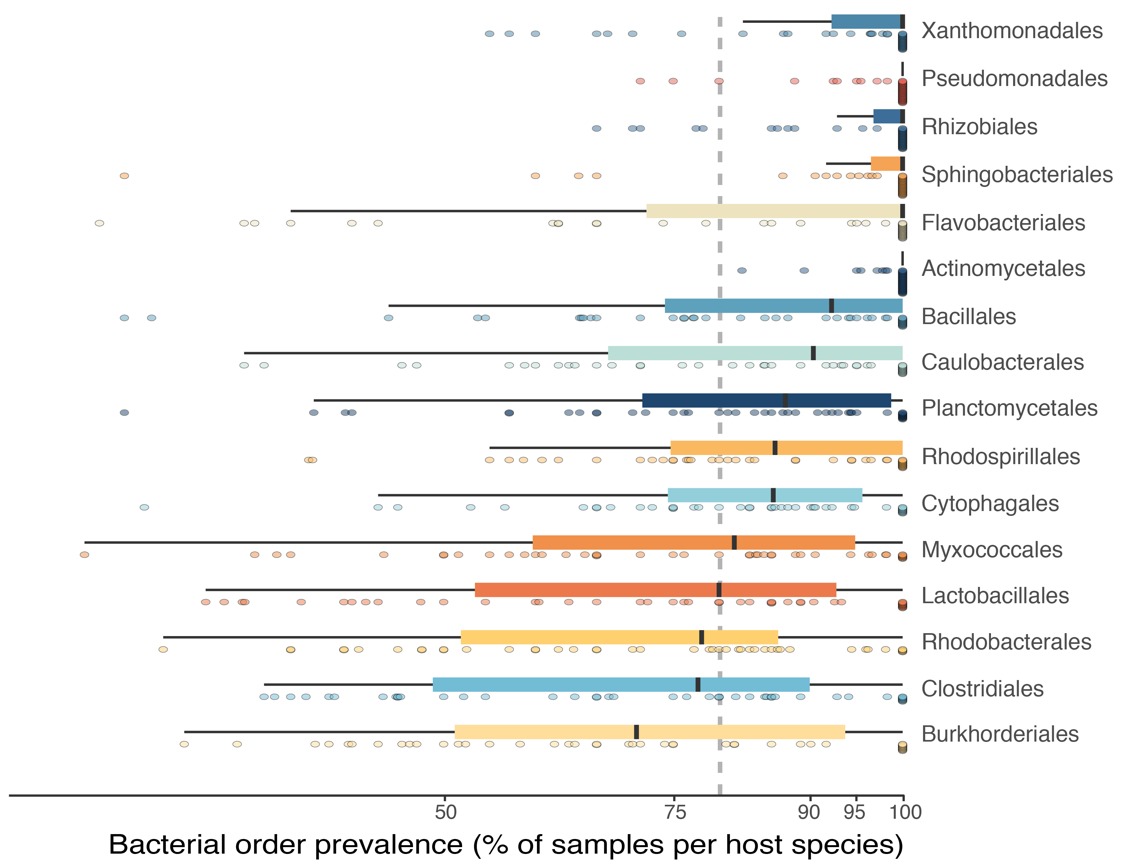
**

**Supplementary fig. S2. Prevalence of skin bacterial taxa (orders) shared among all salamander host species.** Boxplots depict the distribution of estimated prevalence of the 16 shared bacterial orders across salamander host species. Filled circles correspond to bacterial prevalence estimated within each salamander host species.

**
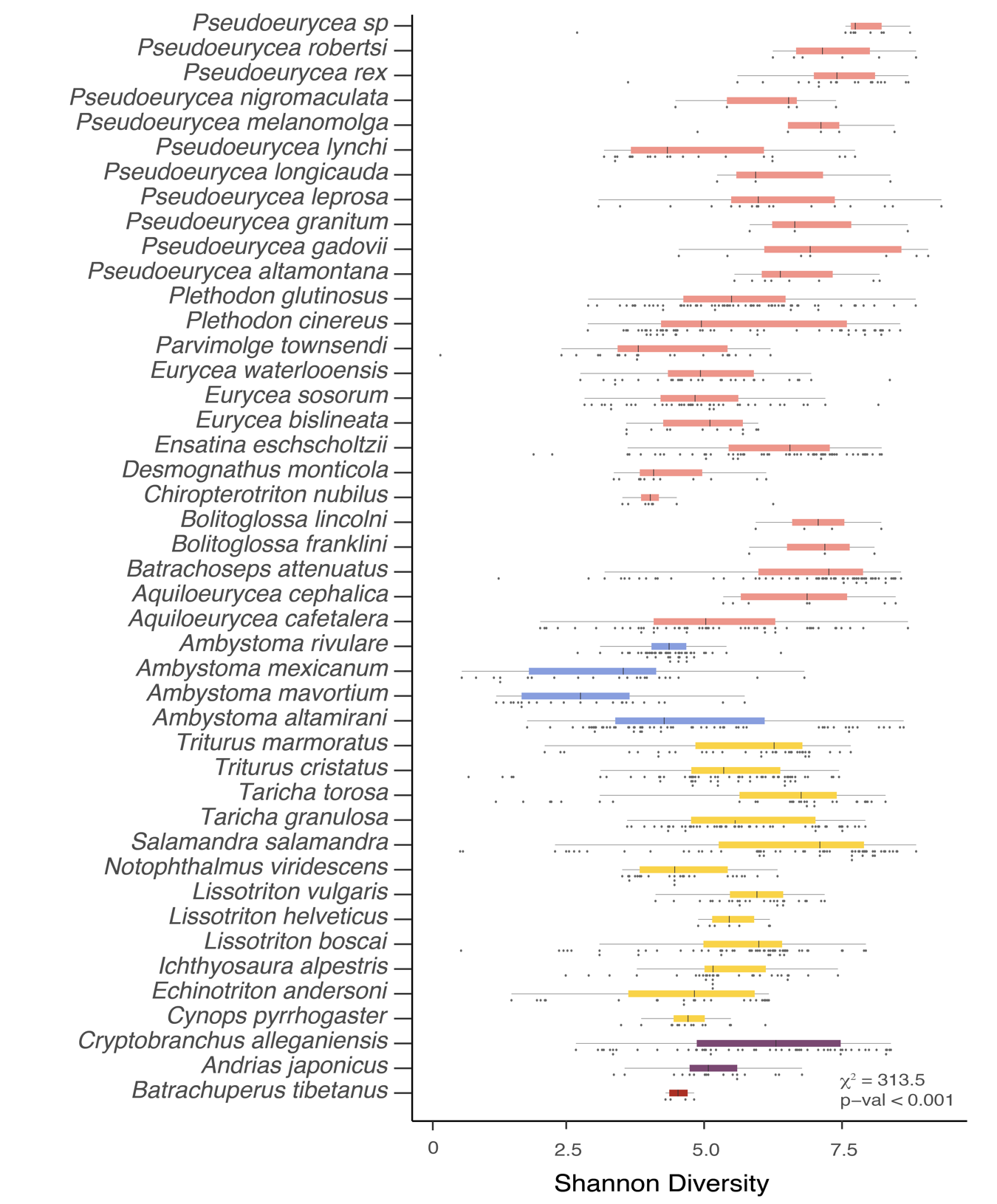
**

**Supplementary fig. S3. Skin microbial diversity of salamander species.** Boxplots depicting the distribution of Shannon’s entropy estimates for skin microbiomes sampled across 41 salamander host species. Filled circles represent Shannon’s entropy estimated for each sampled individual. Salamander species are arranged following the phylogeny presented in figure 1.

**
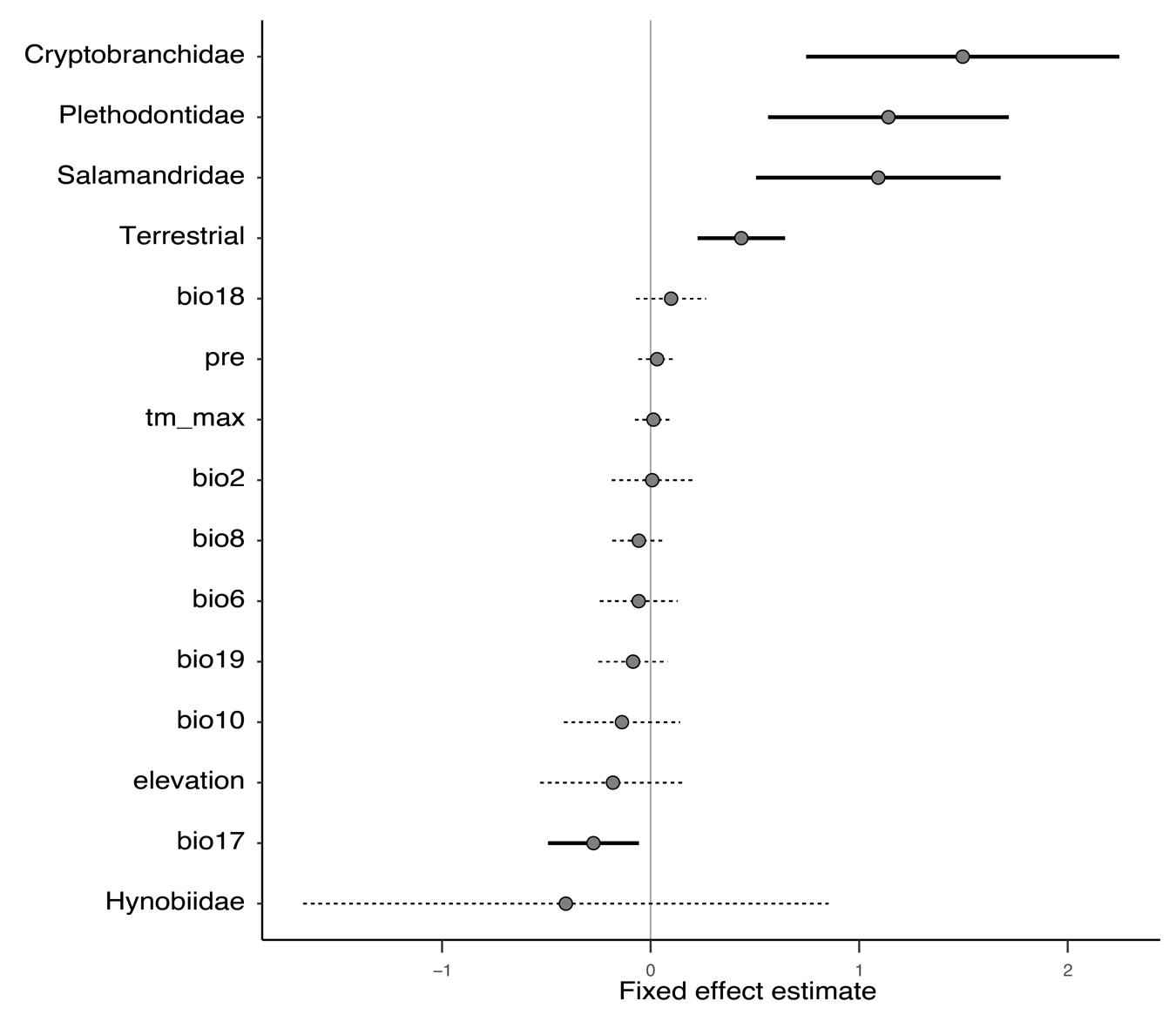
Supplementary fig. S4. Influence of climatic and host factors on the alpha diversity of the salamander skin microbiota.** a, fixed effects of climatic and host factors on alpha diversity estimated using Linear Mixed Models. Horizontal lines represent the confidence interval (95%) for individual fixed effects, with solid and dashed lines indicating significant and non-significant effects, respectively. The reference level for salamander family is Ambystomatidae and the reference level for host habitat is aquatic.

**
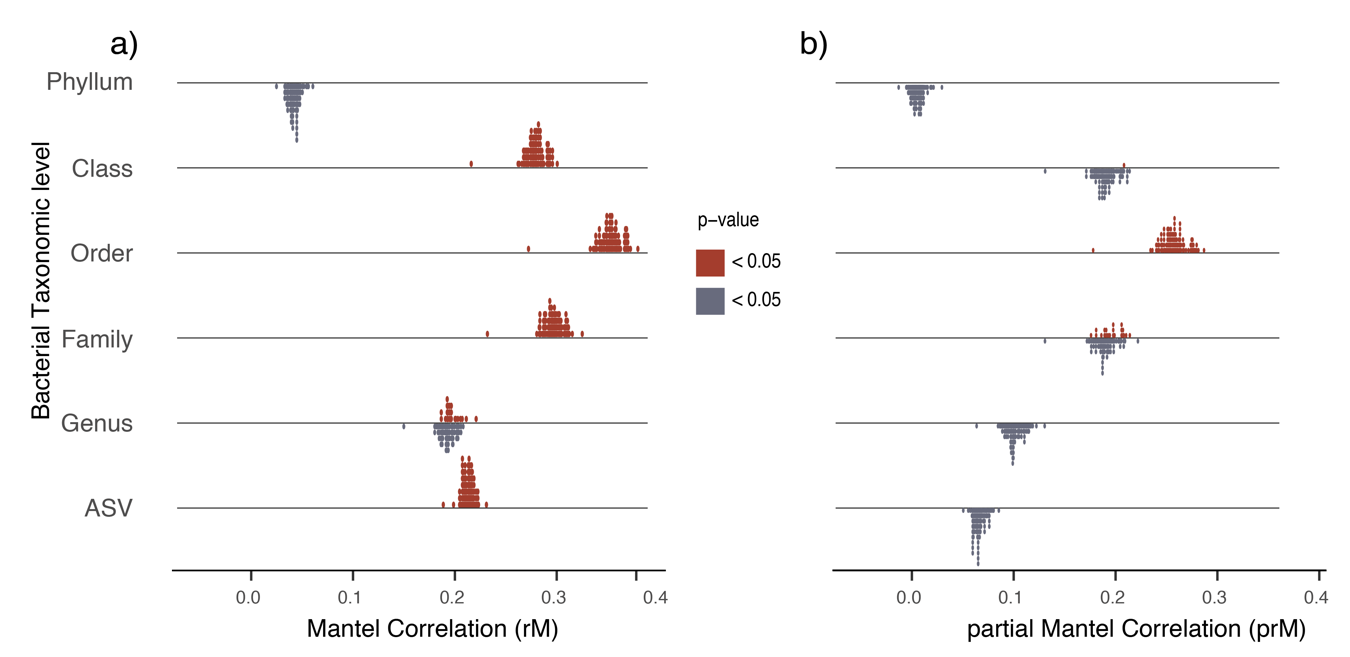
**

**Supplementary fig. S5. Association between salamander phylogenetic distances and skin bacterial community dissimilarity.** Mantel (a) and partial Mantel (b) correlations between host species evolutionary distances and bacterial dissimilarity at different taxonomic levels estimated with 100 bootstrap trees of extant salamanders. Solid circles represent the correlation coefficients estimated for each the bootstrap trees. Colours are indicative of the corresponding p-values of correlations.

**
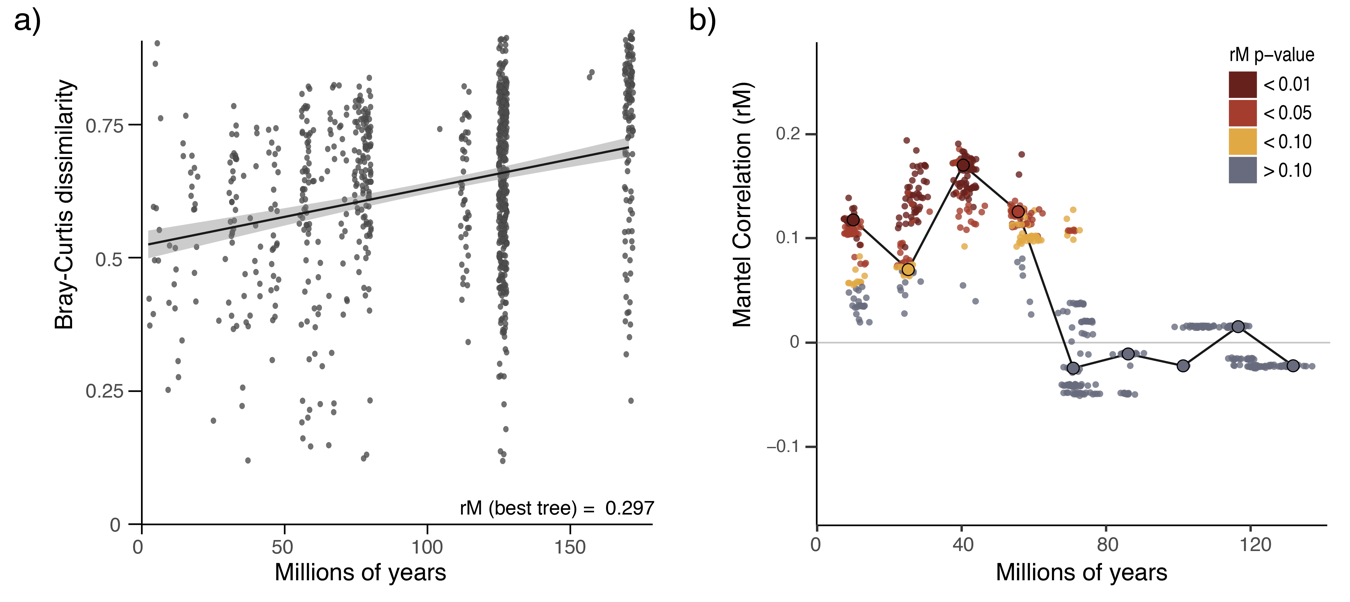
**

**Supplementary fig. S6. Association between salamander phylogenetic distances and skin bacterial community dissimilarity.** a) Bacterial dissimilarity (Y-axis) at the family level as a function of host species evolutionary distances (X-axis) estimated by fossil-based molecular dating of the best-scoring ML tree of extant salamanders. The solid black line represents the slope estimated with a Mantel test between matrices. b) Correlogram showing the variation in the Mantel correlation coefficients as a function of host species evolutionary distances (in millions of years). Open circles connected by a solid black line represent the correlations estimated with the best-scoring ML tree. Solid circles represent the correlations estimated with evolutionary distances using the fossil-based molecular dating of the bootstrap trees. Colours are indicative of the corresponding p-values of correlations.
