# Supplementary material for: Host phylogeny and environment shape the diversity of salamander skin bacterial communities
Source: Anim Microbiome. 2023 Oct 13;5:52. doi: 10.1186/s42523-023-00271-7 (PMC10571319; doi:10.1186/s42523-023-00271-7)
Supplement: Supplementary file 4 — Additional file 4. Fossil calibration list. [file 42523_2023_271_MOESM4_ESM.docx]

**Fossil calibration list used to date the salamander phylogeny of Ramírez-Barahona et al. (2023)**

***†Ambystoma cfmaculatum***

Affinities: Ambystoma (crown)

Period: Latest Miocene – early Pliocene (late Hemphillian)

Calibration age: Zanclean (3.6 mya)

Specimen: ETMNH 8045

Locality: The Gray Fossil Site, Tennessee

Formation: NA

Reference: Darcy (2015)^1^

***†Dicamptodon antiquus***

Affinities: Dicamptodon (stem)

Period: Late Paleocene

Calibration age: Thanetian (56­ mya)

Specimen: UALVP 32387 (Holotype)

Locality: UALVP Smoky Tower #1 locality, West-Central Alberta, Canada

Formation: Paskapoo Fm.

Reference: Naylor & Fox (1993)^2^

***†Seminobatrachus boltyschkensis***

Affinities: Ambystomatoidea (stem)

Period: Late Paleocene–early Eocene

Calibration age: Ypresian (56 mya)

Specimen: PIN 3991/9 (Holotype)

Locality: Drill hole near Boltyshka village, Cherkassy Region, central Ukraine

Formation: Lower unit of unnamed sapropelite strata

Reference: Skutschas & Gubin (2012)^3^

***†Proamphiuma cretaceae***

Affinities: Amphiumidae (stem)

Period: Late Maastrichtian-early Paleocene

Calibration age: Danian (61.6 mya)

Specimen: MCZ 3504 (Holotype)

Locality: Bug Creek Anthills, McCone County, Montana, USA

Formation: Hell Creek Fm.

Reference: Gardner (2003)^4^

***†Cryptobranchus saskatchewanensis***

Affinties: Cryptobranchidae (crown)

Period: Late Paleocene

Calibration age: Thanetian (56 mya)

Specimen: UALVP 14858 (Holotype)

Locality: UALVP Roche Percée loc. UAR 2, southeastern Saskatchewan, Canada

Formation: Ravenscrag Fm.

Reference: Naylor (1981)^5^

***†Palaeoplethodon hispaniolae***

Affinities: Hemidactyliinae (crown)

Period: Late Eocene to Lower Miocene

Calibration age: Burdigalian (15.97 mya)

Specimen: Accession # AM-3-15 (holotype)

Locality: Amber mine in the northern mountain range (Cordillera Septentrional) of the Dominican Republic

Formation: Mamey Group

Reference: Poinar & Wake (2015)^6^

***†Linglongtriton daxishanensis***

Affinities: Hynobiidae (stem)

Period: Upper Jurassic

Calibration age: Oxfordian (157.3 mya)

Specimen: KUP V0277 (Holotype)

Locality: Daxishan fossil locality, ca. 15 km northeast of the county town of Jian-Chang, Liaoning Province, China

Formation: Lanqi/Tiao- jishan Fm.

Reference: Jia & Gao (2019)^7^

***†Habrosaurus prodilatus***

Affinities: Sirenidae (stem)

Period: Middle Campanian

Calibration age: Campanian (72.1 mya)

Specime: UALVP 43906 (Holotype)

Locality: Irvine microvertebrate locality, Alberta, Canada

Formation: Dinosaur Park Fm.

Reference: Gardner (2003)^8^

***†Carpathotriton matraensi***

Affinites: core Molgini (crown)

Period: Middle Miocene (MN 7/8)

Calibration age: Late Serravalian (11.63 mya)

Specimen: MMP.2005.361 (Holotype)

Locality: Matraszolos locality 2, a section of road below the Rakoczi Chapel, situated at the northern margin of the small village of Matraszolos, North Hungary

Formation: Sajo Valley Fm.

Reference: Venczel (2008)^9^

***†Triassurus sixtelae***

Affinities Caudata (stem)

Period: Ladinian/Carnian

Calibration age: Carnian (227 mya)

Specimen: IN-2584/10 (Holotype)

Locality: Madygen, Turkestan Range, southwestern Kyrgyzstan

Formation: Madygen Fm.

Reference: Schoch et al. (2020)^10^

***†Plethodon sp***

Affinities: Plethodon (stem)

Period: Lower Miocene

Calibration age: Burdigalian (15.97 mya)

Specimen: MSUMP 6504-2003

Locality: Granite County, Montana

Formation: Cabbage Patch Fm.

Reference: Tihen & Wake (1981)^11^

***†Kiyatriton leshchinskiyi***

Affinities: Caudata (crown)

Period: Bathonian

Calibration age: Bathonian (166.1 mya)

Specimen: ZIN PH 9/144 (Holotype)

Locality: Berezovsk Quarry, 2 km south of Nikol’skoe village, Sharypovo District, Krasnoyarsk Territory, Western Siberia, Russia

Formation: Grey clays of the upper part of the Itat Formation

Reference: Skutschas (2016)^12^

***†Paranecturus garbanii***

Affinities: Proteidae (stem)

Period: Late Maastrichtian

Calibration age: Maastrichtian (66)

Specimen: UWBM 93370 (Holotype)

Locality: UWBM C1153 (=UCMP V82022), Garfield County, Montana, U.S.A.

Formation: Hell Creek Fm.

Reference: Demar (2013)^13^

***†Chelotriton sp***

Affinities: Pleurodelini (crown)

Period: Latest Oligocene

Calibration age: Chattian (23.03 mya)

Specimen: MB.Am.45

Locality: Orsberg near Erpel, close to Linz in Rhineland-Palatinate, Germany

Formation: Blätterkohle

Reference: Marjanovic & Witzmann (2015)^14^

***†Palaeoproteus gailicus***

Affinities: Salamandridae (crown)

Period: Paleocene

Calibration age: Thanetian (56 mya)

Specimen: C.R. No. 6692

Locality: Cernay, Lemoine Quarry

Formation: Conglomerat de Cernay

Reference: Estes et al. (1967)^15^

***†Taricha oligocenica***

Affinities: Taricha (stem)

Period: Early Oligocene (Ar: 34.85–32.8 mya)

Calibration age: Rupelian (32.8 mya)

Specimen: UOMNH F-5405 (Holotype)

Locality: Lane County, Oregon

Formation: Mehama Fm.

Reference: Jacisin & Hopkins (2018)^16^

***†Xenopus arabiensis***

Affinities: Xenopus (crown)

Period: Late Oligocene

Calibration age: Chattian (23.03 mya)

Specime: AMNH VP 30009 (Holotype)

Locality: Ar Rhyashia in central western Yemen on the highlands west of the Great Escarpment

Formation: Yemen Volcanic Group

Reference: Henrici & Baez (2001)^17^

***†Aneides sp***

Affinities: Aneides (stem)

Period: Lower Miocene

Calibration age: Aquitanian (15.97 mya)

Specimen: MSUMP 6504-2004

Locality: Granite County, Montana

Formation: Cabbage Patch Fm.

Reference: Tihen & Wake (1981)^11^

***†Amphiuma jepseni***

Affinities: Amphiumidae (stem)

Period: Late Paleocene

Calibration age: Thanetian (56 mya)

Specimen: NA

Locality: Princeton Quarry local fauna (Princeton [= Silver Coulee], Fritz, and Schaff quarries), Park County, northwestern Wyoming, USA

Formation: Fort Union Fm.

Reference: Rieppel & Grande (1998)^18^

***†Andrias scheuchzeri***

Affinities: Andrias (stem)

Period: Upper Miocene (MN 7/8)

Calibration age: Late Serravalian (11.63 mya)

Specimen: TSMHN 8, 432 (Lectotype)

Locality: Mataschen/Styria

Formation: NA

Reference: Tempfer-2004

***†Andrias scheuchzeri***

Affinities: Andrias (stem)

Period: Late Miocene–Early Pliocene

Calibration age: Zanclean (3.6 mya)

Specimen: MBFSZ V 2019.99.1., PTE-5152; MBFSZ V.2019.97.1., 98.1.; MBFSZ V.2019.103.1.105.1., 106.1., 107.1., 108.1., 109.1., PTE- 5148–5151, JAM 2015.10.1; MBFSZ V.2019.100.1., 101.1., 102.1., 104.1., 110.1., JAM 2006.217.155, 2008.537.1, T/2019.4.1.; JAM 2006.236.96, MBFSZ V.2019.111.1., 113.1., 114.1.; JAM T/2019.2.1., 3.1., MBFSZ V.2019.112.1.; JAM 2006.185.47; JAM T/2019.1.1.

Locality: SW Hungary, on the SE margin of the Mecsek Mountains, at the eastern boundary of the city of Pécs

Formation: Pécs-Danitzpuszta sand pit

Reference: Szentesi etal-2020

***†Chunerpeton tianyensis***

Affinities: Cryptobranchidae (stem)

Period: Bathonian (Late Jurassic according to Roelant etal-2007)

Calibration age: Tithonian (145 mya)

Specimen: Chinese Academy of Geological Sciences (CAGS)-IG- 02051 (Holotype)

Locality: Daohugou, Ningcheng County, Inner Mongolia, China

Formation: Jiulongshan Fm.

Reference: Gao Shubin-2003

***†Habrosaurus dilatus***

Affinities: Sirenidae (stem)

Period: Late Maastrichtian

Calibration age: Maastrichtian (66 mya)

Specimen: USNM 10749 (Holotype)

Locality: unrecorded locality in type area of formation, Niobrara County, Wyoming, USA

Formation: Lance Fm.

Reference: Gardner (2003)^8^

***†Egoria malashichevi***

Affinities: Caudata (stem)

Period: Middle Jurassic (Bathonian)

Calibration age: Bathonian (166.1 mya)

Specimen: ZIN PH 40/144 (Holotype)

Locality: Berezovsk Quarry, near Nikol’skoe Village, Sharypovo District, Krasnoyarsk Territory, Russia

Formation: upper part of Itat Fm.

Reference: Skutschas et al (2020)^19^

***†Kokartus honorarius***

Affinities: Caudata (stem)

Period: Late Bathonian–Callovian

Calibration age: Callovian (163.5 mya)

Specimen: CCMGE 1/11998 (Holotype)

Locality: Nichke 1, Kugart 1, and Kyzylsu 1, Kyrgystan

Formation: Balabansai Svita

Reference: Skutschas & Martin (2011)^20^

***†Marmorerpeton freemani***

Affinities: Caudata (stem)

Period: Upper Bathonian

Calibration age: Bathonian (166.1 mya)

Specimen: British Museum (Natural History) Department of Palaeontology R.11364 (Holotype)

Locality: Old Cement Works Quarry, Kirtlington, Oxfordshire, England

Formation: Kirtlington Mammal Bed, near base of the Forest Marble

Reference: Evans et al. (1988)^21^

***†Marmorerpeton kermacki***

Affinities: Caudata (stem)

Period: Upper Bathonian

Calibration age: Bathonian (166.1 mya)

Specimen: British Museum (Natural History) Department of Palaeontology R.11361 (Holotype)

Locality: Old Cement Works Quarry, Kirtlington, Oxfordshire, England

Formation: Kirtlington Mammal Bed, near base of the Forest Marble

Reference: Evans et al. (1988)^21^

***†Marmorerpeton sp***

Affinities: Caudata (stem)

Period: Upper Bathonian

Calibration age: Bathonian (166.1 mya)

Specime: BMNH R.11365, R.11366, R.11367, R.11368 (EF 76 : 1 : 11 : 1), R.11369, R.11370 (EF 77 : 5 : 1 : 5); R.11371, R.11372, R.11373, R.11374, R.11375, R.11376, R.11377, R. 11378, R.11379 (EF 77 : 1 : 1 : 1), R.11380

Locality: Old Cement Works Quarry, Kirtlington, Oxfordshire, England

Formation: Kirtlington Mammal Bed, near base of the Forest Marble

Reference: Evans et al. (1988)^21^

***†Pangerpeton sinensis***

Affinities: Caudata (stem)

Period: Late Jurassic–Early Cretaceous

Calibration age: Tithonian (145 mya)

Specimen: IVPP V14244 (Holotype)

Locality: Wubaiding Village, Lingyuan City, Liaoning Province, China

Formation: Contemporaneous with the salamanderbearing horizon at Daohugou, Inner Mongolia

Reference: Wang & Evans (2006)^22^

***†Triadobatrachus massinoti***

Affinities: Anura (stem)

Period: Gyronitian

Calibration age: Induan (251.2 mya)

Specimen: MNHN, nº MAE 126 (Holotype)

Locality: Betsieka, Madagascar

Formation: equated to the Sakamena group

Reference: Rage & Rocek (1989)^23^

***†Urupia monstrosa***

Affinities: Caudata (stem)

Period: Bathonian

Calibration age: Bathonian (166.1 mya)

Specimen: ZIN PH 1/144 (Holotype)

Locality: Berezovsk Quarry, 2 km south of Nikol’skoe village, Sharypovo District, Krasnoyarsk Territory, Western Siberia, Russia

Formation: Grey clays of the upper part of the Itat Formation

Reference: Skutschas & Krasnolutskii (2011)^24^

***†Balveherpeton hoennetalensis***

Affinnities: Salamandroidea (stem)

Period: Barremian–Aptian

Calibration age: Aptian (113 mya)

Specimen: WMNM P76313 (Holotype)

Locality: Balve locality

Formation: Fissure fillings within the Middle to Upper Devonian reef limestone (Massenkalk)

Reference: Skutschas et al. (2020)^25^

***†Valdotriton gracilis***

Affinnities: Salamandroidea (stem)

Period: Early Barremian

Calibration age: Barremian (125 mya)

Specime: LH 928061a-b (Holotype)

Locality: Las Hoyas fossil site, La Cierva township, about 20km east of Cuenca, Cuenca Province, Castilla La Mancha, Spain

Formation: Limestone in Unit III of the La Huérguina Fm.

Reference: Evans & Milner (2006)^26^

***†Iridotriton hechtii***

Affinnities: Salamandroidea (stem)

Period: Kimmeridgian–Early Tithonian

Calibration age: Tithonian (145 mya)

Specimen: DINO 16453a (Holotype)

Locality: Dinosaur National Monument, Rainbow Park microsite (Dinosaur National Monument no. 96), Utah, USA

Formation: Brushy Basin Member of the Morrison Fm.

Reference: Evans et al. (2005)^27^

***†Koaliella sp***

Affinnities: Salamandroidea (crown)

Period: Paleocene

Calibrarion age: Thanetian (56 mya)

Specimen: C.R. No. 6740

Locality: Cernay, Lemoine Quarry

Formation: Conglomerat de Cernay

Reference: Estes et al. (1967)^15^

***†Koalliella genzeli***

Affinities: Salamandridae (crown)

Period: NA

Calibration age: NA

Specimen: NA

Locality: NA

Formation: NA

Reference: Estes (1981)^28^

***†Phosphotriton sigei***

Affinities: Salamandridae (crown)

Period: Likely Eocene (late Middle or Late Eocene)

Calibration age: Eocene (33.9 mya)

Specimen: MNHN.F.QU17755 (Holotype)

Locality: Unknown locality of the Phosphorites du Quercy, southwestern France

Formation: Phosphorites du Quercy

Reference: Tissier et al. (2016)^29^

***†Taricha lindoei***

Affinities: Taricha (stem)

Period: Early Oligocene (Ar: 39–30 mya)

Calibration age: Rupelian (30 mya)

Specimen: UALVP 13870 (Holotype)

Locality: Wheeler County, Oregon.

Formation: Big Basin Member, John Day Fm.

Reference: Jacisin & Hopkins (2018)^16^

**References**

1. Darcy, H. E. Additional Research and Taxonomic Resolution of Salamanders ( Amphibia : Caudata ) from the Mio- Pliocene Gray Fossil Site , TN. (East Tennessee State University, 2015).

2. Naylor, B. G. & Fox, R. C. A new Ambystomatid salamander, Dicamptodon antiquus n.sp., from the Paleocene of Alberta, Canada. Canadian Journal of Earth Sciences. 30(4): 814-818. *Can. J. Earth Sci.* **30**, 814–818 (2011).

3. Skutschas, P. P. & Gubin, Y. M. A new salamander from the late Paleocene – early Eocene of Ukraine. *Acta Paleontol. Pol.* **57**, 135–148 (2012).

4. Gardner, J. D. The fossil salamander Proamphiuma cretacea Estes ( Caudata ; Amphiumidae ) and relationships within the Amphiumidae. *J. Vertebr. Paleontol.* **23**, 769–782 (2003).

5. Naylor, B. G. Cryptobranchid salamanders from the Paleocene and Miocene of Saskatchewan. *Copeia* **1981**, 76–86 (1981).

6. Poinar, G. & Wake, D. B. Palaeoplethodon hispaniolae gen. n., sp. n. (Amphibia: Caudata), a fossil salamander from the Caribbean. *Palaeodiversity* **8**, 21–29 (2015).

7. Jia, J. & Gao, K. A new stem hynobiid salamander (Urodela, Cryptobranchoidea) from the Upper Jurassic (Oxfordian) of Liaoning Province, China. *J. Vertebr. Paleontol.* **39**, e1588285 (2019).

8. Gardner, J. D. Revision of Habrosaurus Gilmore(Caudata; Sirenidae) and relationships among sirenid salamanders. *Palaeontology* **46**, 1089–1122 (2003).

9. Venczel, M. A new salamandrid amphibian from the Middle Miocene of Hungary and its phylogenetic relationships. *J. Syst. Palaeontol. ISSN* **6**, 41–59 (2008).

10. Schoch, R. R., Werneburg, R. & Voigt, S. A Triassic stem-salamander from Kyrgyzstan and the origin of salamanders. *Proc. Natl. Acad. Sci.* **117**, (2020).

11. Tihen, J. A. & Wake, D. B. Vertebrae of Plethodontid Salamanders from the Lower Miocene of Montana. *J. Herpetol.* **15**, 35–40 (1981).

12. Skutschas, P. P. A new crown-group salamander from the Middle Jurassic of Western Siberia , Russia. *Palaeobiodiversity and Palaeoenvironments* **96**, 41–48 (2016).

13. Demar, D. G. J. A new fossil salamander ( Caudata , Proteidae ) from the Upper Cretaceous ( Maastrichtian ) Hell Creek. *J. Vertebr. Paleontol. ISSN* **33**, 588–598 (2013).

14. Marjanovic, D. & Witzmann, F. An Extremely Peramorphic Newt ( Urodela : Salamandridae : Pleurodelini ) from the Latest Oligocene of Germany , and a New Phylogenetic Analysis of Extant and Extinct Salamandrids. *PLoS One* **10**, e0137068 (2015).

15. Estes, R., Hecht, M. & Hoffstetter, R. Paleocene amphibians from Cernay, France. *Am. Museum Novit.* **2295**, 1–26 (1967).

16. Jacisin, J. J. & Hopkins, S. S. B. A redescription and phylogenetic analysis based on new material of the fossil newts Taricha oligocenica Van Frank, 1955 and Taricha lindoei Naylor, 1979 (Amphibia , Salamandridae) from the Oligocene of Oregon. *J. Paleontol.* **92**, 713–733 (2018).

17. Henrici, A. C. & Báez, A. M. First occurrence of Xenoipus (Anura: Pipidae) on the Arabian Peninsula: a nes species from the upper Oligocene of Yemen. *J. Paleontol.* **75**, 870–882 (2001).

18. Rieppel, O. & Grande, L. A well-preserved fossil amphiumid (Lissamphibia: Caudata) from the Eocene Green River Formation of Wyoming. *J. Vertebr. Paleontol.* **18**, 700–708 (1998).

19. Skutschas, P. *et al.* A new small-sized stem salamander from the Middle Jurassic of Western Siberia , Russia. *PLoS One* **15**, e0228610 (2020).

20. Skutschas, P. & Martin, T. Cranial anatomy of the stem salamander Kokartus honorarius ( Amphibia : Caudata ) from the Middle Jurassic of Kyrgyzstan. *Zool. J. Linn. Soc.* **161**, 816–838 (2011).

21. Evans, S. E., Milner, A. R. & Mussett, F. The earliest known salamanders (Amphibia, Caudata): a records from the Middle Jurassic of England. *Geobios* **21**, 539–552 (1988).

22. Wang, Y. & Evans, S. E. A new short−bodied salamander from the Upper Jurassic/Lower Cretaceous of China. *Acta Paleontol. Pol.* **51**, 127–130 (2006).

23. Rage, J. & Rocek, Z. Redescription of Triadobatrachus massinoti (Piveteau, 1936) an anuran amphibian from the Early Triassic. (1989). *Palaeontogr. Abt. A* **206**, 1–16 (1989).

24. Skutschas, P. P. & Krasnolutskii, S. A. A new genus and species of basal salamanders from the Middle Jurassic of Western Siberia, Russia. *Proc. Zool. Inst. RAS* **315**, 167–175 (2011).

25. Skutschas, P. P., Kolchanov, V. V & Schwermann, A. H. First salamander from the Lower Cretaceous of Germany. *Cretac. Res.* **116**, 104606 (2020).

26. Evans, S. E. & Milner, A. R. A metamorphosed salamander from the early Cretaceous of Las Hoyas, Spain. *Philos. Trans. R. Soc. B* **351**, 627–646 (1996).

27. Evans, S. E., Lally, C., Chure, D. C., Elder, A. & Maisano, J. A. A Late Jurassic salamander (Amphibia: Caudata) from the Morrison Formation of North America. *Zool. J. Linn. Soc.* **143**, 599–616 (2005).

28. Estes, R. *Handbuch der Paläoherpetologie/Encyclopedia of Paleoherpetology. Part 2. Gymnophiona, Caudata*. (Gustav Fischer Verlag, 1981).

29. Tissier, J. *et al.* Synchrotron analysis of a ‘mummified’ salamander (Vertebrata: Caudata) from the Eocene of Quercy, France. *Zool. J. Linn. Soc.* **177**, 147–164 (2016).
